# Mapping susceptibility loci for alcohol consumption using number of grams of alcohol consumed per day as a phenotype measure

**DOI:** 10.1186/1471-2156-4-S1-S104

**Published:** 2003-12-31

**Authors:** Jennie Z Ma, Dong Zhang, Randolph T Dupont, Michael Dockter, Robert C Elston, Ming D Li

**Affiliations:** 1 Department of Psychiatry, The University of Texas Health Science Center at San Antonio, San Antonio, Texas, USA; 2 Departments of Psychiatry and Medicine, The University of Tennessee Health Science Center, Memphis, Tennessee, USA; 3 Department of Epidemiology and Biostatistics, Case Western University, Cleveland, Ohio, USA

## Abstract

**Background:**

There is substantial evidence for a significant genetic component to the risk for alcoholism. However, susceptibility loci or genes for alcohol dependence remain largely unknown. To identify susceptibility loci for alcohol dependence, we selected 329 extended families from the Framingham Heart Study population in which at least one family member reported alcohol consumption during the interview in 1970–1971, and performed genome-wide linkage analyses using various analytical methods.

**Results:**

Multi-point sib-pair regression analysis using the SIBPAL program of S.A.G.E. provided strong evidence for linkage of alcohol dependence to chromosomes 9 (p-value < 0.0001) and weak evidence to chromosomes 15 and 16 (p-value < 0.005). To confirm these findings, we re-analyzed the same data set by various methods implemented in GENEHUNTER and found that only one region was significant with a LOD score > 2.0 by the variance-component method. This region is located on chromosome 9 between markers GATA21F05 and GATA81C04.

**Conclusion:**

Analyses of the Framingham Heart Study population provided evidence of genetic susceptibility loci for alcohol dependence on chromosomes 9, 15, and 16. The genomic region identified on chromosome 9 was particularly interesting because the region has also been previously reported to be linked to alcohol dependence in the American Indian population by another group.

## Background

Many twin, family, and adoption studies have provided strong evidence of the important role of genes and environment in the etiology of alcoholism in both men [1] and women [2]. The heritability of alcohol dependence was estimated to be approximately 60% in alcohol users [3]. The specific genetic factors underlying susceptibility to alcoholism remain, however, largely unknown. To identify susceptibility loci for alcohol dependence and related phenotypes, several studies have been conducted and reported in the literature (e.g., [4-7]). However, apart from the susceptibility loci for alcohol dependence on chromosome 4 that was identified by three studies, no other region has been confirmed in more than one population. To further confirm these earlier reports and identify any additional susceptibility loci for alcohol dependence, in this study we performed a genome-wide scan in the Framingham Heart Study population using the number of grams of alcohol consumed per day as an alcoholism phenotype.

## Methods

A total of 330 extended pedigrees from the original and offspring cohorts of the Framingham Heart Study were provided through the Genetic Analysis Workshop 13 (GAW13). Of these, 329 families had at least one family member who reported alcohol consumption during 1970–1971. These 329 families were used for a genome-wide scan to identify susceptibility loci for alcohol dependence. The measurement of alcohol dependence in our study was based on self-reported number of grams of alcohol consumed per day during 1970–1971. The alcohol measure in the number of grams of alcohol consumed per day was highly skewed and its distribution differed between men and women. It is likely that the extremely high values of alcohol consumption were inflated, especially for male alcohol users. To minimize the impact of such inflation on linkage analysis results, and to reduce the skewness due to individuals who reported extremely high alcohol consumption per day, we transformed the number of grams of alcohol consumed per day into natural logs prior to linkage analysis. For individuals (*N *= 502) who reported zero alcohol consumption, we set their phenotypes equal to zero and included them in the analysis. Skewness and kurtosis of this log-transformed alcohol consumption per day were 0.144 and 1.97, respectively. If we excluded individuals who reported no alcohol consumption during 1970–1971, skewness and kurtosis became 0.193 and 2.17, respectively.

Linkage analyses were performed using the programs GENIBD and SIBPAL of S.A.G.E. (version 4.2) and GENEHUNTER (version 2.1; [8]). Multi-point IBD sharing for interval scanning was generated for full-sib pairs using GENIBD. In SIBPAL, the weighted combination of squared trait differences and squared mean-corrected trait sums, adjusting for the non-independence of sib pairs (defined in SIBPAL as the w3 option) and for the non-independence of squared trait sums and differences (defined as the w4 option), were used as the dependent variables in the trait regression analysis [9]. The options w3 and w4 yielded essentially the same results. Sex and age were included as covariates for some analyses; however, no significant differences were obtained between the final mapping results when compared with those without adjustment for sex and age. The S-PLUS 6.1 and SAS 8.2 packages were used to prepare the data in the required format and to summarize the results generated from these linkage analyses.

## Results

Table 1 summarizes some clinical characteristics of the samples used in the study. A total of 4681 subjects from 329 extended families were included and 2565 of them had alcohol consumption information available. Of these, 1698 were genotyped for 401 markers at an average spacing of 7.5 cM between markers. There were about 14 members per family, who had an average age of 43.14 with standard deviation 16.70. Among the 2063 alcohol users, there exists a substantial variation in the number of grams of alcohol consumed per day, with a range of 2 to 537 g for male and 2 to 139 g for female alcohol users. To reduce the skewness of alcohol consumption per day and to minimize the impact of extremely inflated high values of alcohol consumption on the linkage analysis, the number of grams of alcohol consumed per day was natural log-transformed, when greater than zero, and used as the phenotypic measure for alcohol consumption.

**Figure 1:**
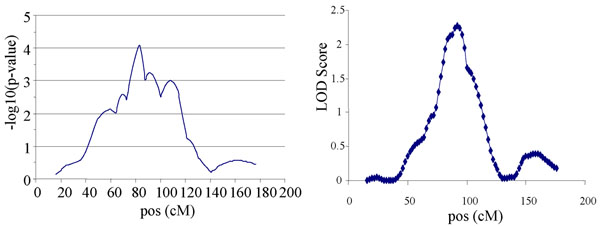
Linkage analysis results for chromosome 9 using the model-free sib pair multi-point regression method of S.A.G.E. (left panel) and the variance component method of GENEHUNTER (right panel).

Model-free multi-point sib-pair regression analysis using the SIBPAL program of S.A.G.E. with both the w3 and w4 options provided evidence for linkage of alcohol dependence to a genomic region on chromosome 9 at a significance level of 0.0001, and on chromosomes 15 and 16 at a significance level of 0.005. Table 2 summarizes the sib-pair regression results with approximate chromosome locations from the SIBPAL program of S.A.G.E. with the w3 option. The left panel of Figure 1 further depicts the negative log *p*-values to base 10 for chromosome 9.

When we performed genome-wide scans using various methods implemented in GENEHUNTER, one region, located on chromosome 9 between markers GATA21F05 and GATA81C04, was identified by the variance-component method with a maximal LOD score of 2.27 (see the right panel of Figure 1).

## Discussion

In this study, by using the model-free multi-point sib-pair regression method, we found evidence for linkage of the number of grams of alcohol consumed per day to chromosomes 9, 15, and 16. To confirm these results, we also carried out a genome-wide scan using various methods implemented in GENEHUNTER. Only one susceptibility locus, on chromosome 9 between the markers GATA21F05 and GATA81C04, was identified by the variance-component method. This represents the same region identified by multi-point sib-pair regression analysis.

Alcohol dependence is a complex disorder, which is determined by both genetic and environmental factors. To identify susceptibility loci for alcohol dependence and related phenotypes, Reich and his colleagues [4] genotyped 225 sib pairs for 291 markers followed by model-free two-point and multi-point regression analyses. Their results provided strong evidence for linkage of alcohol dependence to genomic regions on chromosomes 1 and 7, a more modest evidence for a locus on chromosome 2, and suggestive evidence for a locus near the alcohol dehydrogenase genes on chromosome 4. In an independent study, Long et al. [5] scanned 172 sib pairs for 517 markers in an American Indian population and found that the best evidence is obtained with marker D11S1984 on chromosome 11, and there was good evidence on chromosome 4 at the D4S3242 locus. Using the same criteria as employed by Reich et al. [4], Foroud et al. [6] genotyped an additional 266 sib pairs for 351 markers. Their results further confirmed the linkage of alcohol dependence to chromosomes 1 and 7. Additionally, they found more susceptibility loci on chromosomes 2 and 3. Using the maximum number of drinks consumed in a 24-hour period as an alcoholic phenotype, Saccone et al. [7] found strong evidence for linkage to chromosome 4, which is consistent with previous reports [4,5]. However, no linkage at a significant level of 0.01 or a LOD score greater than 1.18 was detected in our study for these previously reported positive regions for alcohol dependence. In this study, we used self-reported number of grams of alcohol consumed per day as an alcoholism phenotype for our linkage analysis. In our view, this measure may be more or less similar to the 'maximum number of drinks consumed in a 24-hour period' used by Saccone et al. [7] in the Collaborative Study on the Genetics of Alcoholism. This phenotype may represent one of the simplest measures of alcohol dependence based on self-reported interview items. The relationship between the number of grams of alcohol consumed per day and diagnosis of alcoholism remains to be investigated. Since the former was the only information on alcoholism available in the Framingham Heart Study data set provided through GAW13, we used it in our linkage analysis.

The possibility of a susceptibility locus for alcohol dependence on chromosome 9 is of interest and warrants further investigation. This region was detected by both model-free multi-point sib-pair regression analysis with a *p-*value of 0.00008 and variance component analysis with a maximal LOD score of 2.27. By using as the alcoholic phenotype the maximum reported number of grams of alcohol consumed per day from Exams 1, 2, 4, and 7 of the original cohort and Exams 1 to 4 of the offspring cohort, or from all exams of both cohorts, Bergen et al. [10] also found evidence for linkage of alcohol consumption to the same region on chromosome 9 with a *p*-value of 3.77x10^-4^. In an independent study of American Indian population, Long et al. [5] found modest evidence of linkage of alcohol dependence to this region (*p*-value = 0.00958). The presence of a positive region on chromosome 9 with a significant *p*-value or LOD score in the Framingham Heart Study population (predominantly Caucasian Americans), coupled with the putative positive results in the American Indian population, suggests that this region may harbor susceptibility gene(s) for alcohol dependence. On the basis of the human genome draft sequence, we predict that approximately 40 genes are located within this chromosomal region. Further fine mapping and molecular characterization of these genes are essential to determine which gene(s) may represent a candidate(s) for alcohol dependence.

## Conclusions

Our genome-wide scan results using multi-point sib-pair regression analyses provide evidence for linkage of alcohol consumption to chromosomes 9, 15, and 16. The same locus on chromosome 9 was also identified by the variance component method. Given that the locus on chromosome 9 is linked to different alcohol dependence-related phenotypes in two ethnic populations, it is highly likely that the locus may harbor susceptibility gene(s) for alcohol dependence. Thus it will be important to replicate this finding in future studies.

## Figures and Tables

**Table 1:** Characteristics of extended families used in linkage analysis to identify loci for the number of grams of alcohol consumed per day.

**Category**	**Sample size**^A^	**Mean ± SEM (Range)**
No. of subjects per family	329	14.23 ± 0.46 (7–84)
No. of drinkers per pedigree	329	6.27 ± 0.24 (1–46)
Age of subjects	2565	43.14 ± .33 (5–83)
Male subjects	1233	42.23 ± 0.49 (11–82)
Female subjects	1332	43.97 ± 0.46 (5–83)
Age of drinkers	2063	42.85 ± 0.35 (13–81)
Male drinkers	1047	42.46 ± 0.50 (13–81)
Female smokers	1016	43.25 ± 0.50 (13–80)
No. of grams of alcohol consumed per day	2063	18.02 ± .54 (2–537)
Male drinkers	1047	24.96 ± 0.94 (2–537)
Female drinkers	1016	10.87 ± 0.40 (2–139)

^A^The sample sizes in the first two rows are numbers of pedigrees and those in the rest of the table are numbers of individual subjects.

**Table 2:** Genomic locations linked to alcohol consumption with a *p*-value < 0.005 across the human genome, as detected by sib-pair analysis using the program SIBPAL (with the w3 option).

**Chromosome**	**Locus(s)**	**Map Position (cM)**	***p*-Value**
9	GATA89A11	83.2	0.00008
15	248vc5	21.1	0.0011
16	GATA11C06	136.7	0.0045
